# Polypharmacy in Middle-European Rheumatoid Arthritis-Patients: A Retrospective Longitudinal Cohort Analysis With Systematic Literature Review

**DOI:** 10.3389/fmed.2020.573542

**Published:** 2020-11-19

**Authors:** Jacqueline Désirée Jack, Rick McCutchan, Sarah Maier, Michael Schirmer

**Affiliations:** ^1^Clinic II, Department of Internal Medicine, Medical University Innsbruck, Innsbruck, Austria; ^2^Department of Medical Statistics, Informatics and Health Economics, Medical University Innsbruck, Innsbruck, Austria

**Keywords:** arthritis (including rheumatoid arthritis), clinical pharmacology, polypharmacy (source: MeSH, NML), coding, comorbidities, drug intake, gender differences

## Abstract

**Objective:** To assess polypharmacy and related medication aspects in Middle-European rheumatoid arthritis (RA) patients, and to discuss the results in view of a systematic literature review.

**Methods:** In this retrospective cohort study, charts were reviewed from RA-patients consecutively recruited between September 27, 2017 and April 29, 2019. Drugs were assigned to the Anatomical Therapeutic Chemical (ATC) groups as proposed by the World Health Organization (WHO). Results were compared to those of a systematic literature review.

**Results:** One hundred seventy-five consecutive RA-patients were included. The mean number of drugs was 6.6 ± 3.5, with 2.4 ± 1.2 drugs taken specifically for RA—compared to 2.6 in the literature. 33.7% of patients experienced polypharmacy defined by ≥5 drugs, compared to 61.6% in the literature–with women affected more frequently than men. After 7 years of follow-up, the number of drugs increased in all ATC-groups by an average of 12.7 %, correlating with age (Corrcoeff = 0.46) and comorbidities (Corrcoeff = 0.599). In the literature, polypharmacy is not always defined precisely, and has not been considered in management guidelines so far.

**Conclusion:** Polypharmacy is a frequent issue in RA-management. With an increasing number of comorbidities during the course of the disease, polypharmacy becomes even more relevant.

## Introduction

Several definitions exist for polypharmacy (1), including the number of medications (usually above 4) and their inappropriateness (2, 3). The number of patients affected by polypharmacy considerably varies when using different definitions (3). Polypharmacy may result in unwanted drug interactions (4), and/or lead to an increase of adverse and serious adverse events, with more frequent admissions to hospitals, thus extending the costs of health care (5). In elderly people over 65 years, more than 50% are prescribed more than 6 medications, and almost 20% receive an inappropriate drug (5). An increasing amount of comorbidities is directly linked to the number of medications advised (6). Especially elderly patients are affected and more endangered by the prescription of unnecessary medications due to their usual higher number of comorbidities (7).

Over the last decades, the incidence of rheumatoid arthritis (RA) remained constant but shifted over the years with a decrease in seropositive and an increase of seronegative RA (8). In RA, life expectancy is shortened by 2–3.5-fold compared to the general population (9). Modern treatment medications and approaches enable to lower disease activity, but mortality still remains unchanged higher than in control groups (10). The increased standardized mortality ratio of 50%–independent from age and gender (11)—is related to cardiovascular events, infections, extra-articular manifestations, with a possible (but still not clearly defined) role of treatments such as glucocorticoids. The excess in cardiovascular risk is not fully explained by conventional risk factors like age and arterial hypertension. Comorbidities can decrease life quality and physical functioning and are even important in patients close to remission (12), other comorbidities might not affect or be affected by RA at all (13). Since comorbidities usually need additional treatment, patients with comorbidities are expected to be more exposed to polypharmacy than those patients without comorbidities. Some diseases seem to appear more likely before RA diagnosis and might predispose for RA, such as other autoimmune diseases and epilepsy. Before diagnosis, RA patients do not have more comorbidities than controls (7).

According to a systematic literature review non-compliance is reported in up to 55% of elderly patients with polypharmacy (14), although the percentages of elderly US veterans feeling to have too many medications are surprisingly low with 4% (15). This discrepancy may be result from different definitions of treatment in administrative and clinical settings.

This observational longitudinal cohort-study retrospectively assessed polypharmacy and comorbidities in consecutive Middle-European RA-patients to estimate the frequency and to identify possible causes of polypharmacy, and to discuss local data with results of a systematic literature review (SLR).

## Patients and Methods

### Literature Review

**Figure 1:**
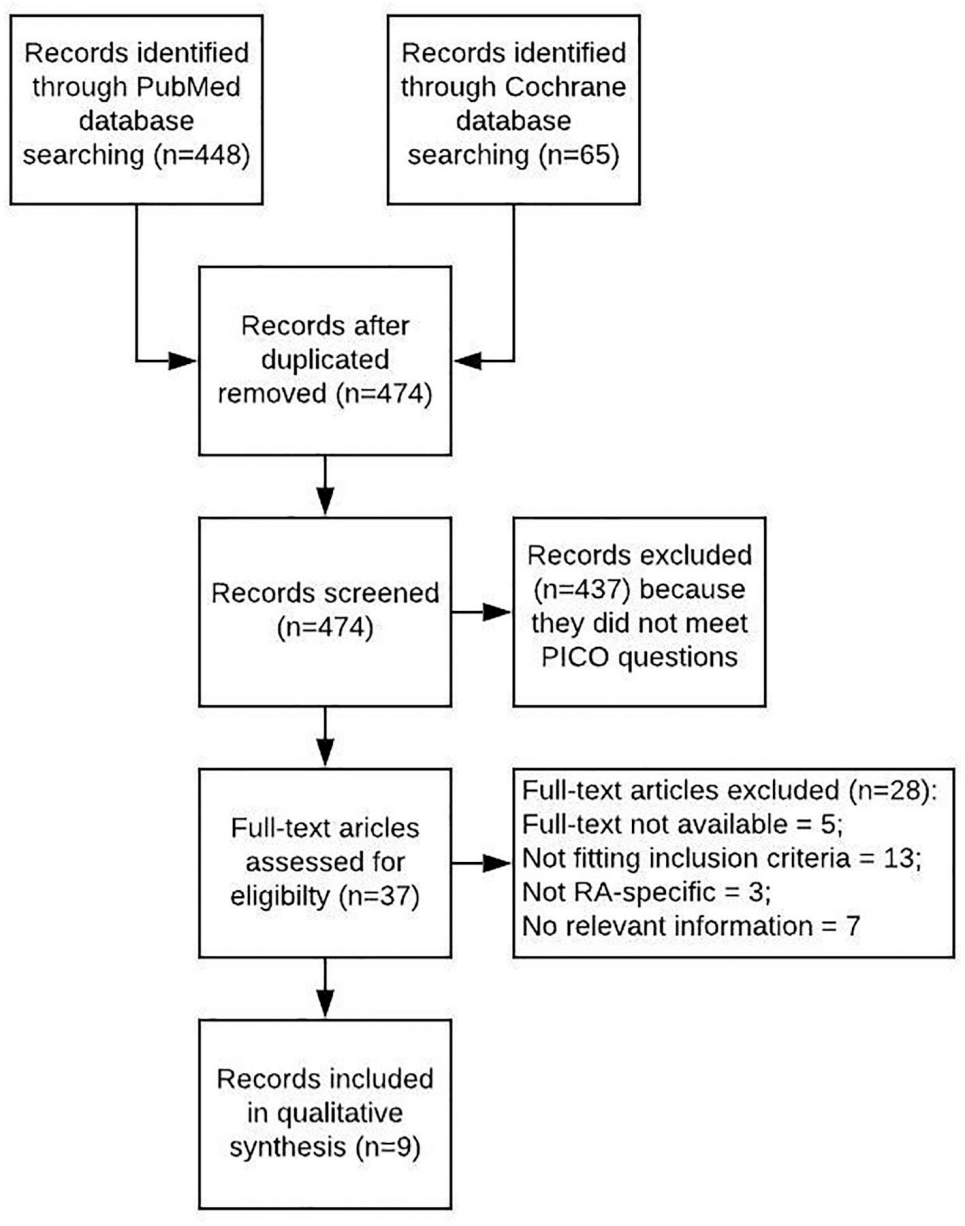
Flowchart picturing the process of the SLR.

A literature review was performed with PICO questions on polypharmacy “as defined by number of medications,” “as defined by number of inappropriate medications according to Beers criteria from 2012” and “depending on number of comorbidities” in RA compared to controls. Search items are listed in the (Appendix 1–3). Publications were included with patients diagnosed with RA, without geographical limits and in English language up to February 2020. Both risk factors and indicators for higher prevalence of polypharmacy were considered as outcome of observational studies published until February 2020. Literature from PubMed and the Cochrane databases was included if written in English language. Additional hand searches were performed in the publications cited for this work. The PRISMA guidelines were applied, Mendeley Desktop (Version 1.19.3) used for citation purposes. Search items are listed in Table 1, and the selection process summarized in a flow diagram (Figure 1).

**Table 1:** Study characteristics from the literature review with Beers criteria as available (ordered according to year of publication).

**Publi-cation**	**Study size**	**Study design**	**Female (%)**	**Age (years)**	**Disease duration**	**Beers criteria**	**Number of comorbidities**	**Refernces**
1985	108 clinical + 153 outpatient	CS	68.8 % clinical 73.7 % outpatient	N/A	N/A	N/A	N/A	(16)
1999	1975: 148 1995: 164	C	1975: 79.1 % 1995: 76.8 %	1975: 46.3 y 1995: 48.6 y	14 y 13.3 y	N/A	1975: 10 % 1995: 15 %	(17)
2007	348	C	71.8%	61.4 y	13.1 y	N/A	Mean 2, 17.2 % 1 21.8 % 2 21.3 % 3 27 % > 3	(6)
2011	295 (~50 % RA)	C	55.6 % >65 y 67.3 % <65 y	73 >65 y 49 <65 y	N/A	N/A	N/A	(18)
2016	54	CS	100%	Without PP: 39 y with PP: 45 y	3 y	N/A	Without PP: 43.3 % with PP: 76.2 %	(19)
2017	1,101	C	78.8%	61.3	10.4 y	N/A	N/A	(20)
2019	200	CS	86%	64	N/A	12x inappropriate-4.2 % 2 duplications-0.7 % 3 contraindications −1 % 2× missing-0.7%	3.1 comorbidities (56.5 % ≥ 3 comorbidities)	(21)
2019	792	CS	89%	56.6	12.7 y	N/A	59 % 1–3 24.5% >3	(22)
2019	22,005	C	76%	57	10	N/A	N/A	(23)

*C, cohort study; CS, cross-sectional study; N/A, not assessed; y, years*.

### Cohort Study and Chart Review

The study is designed as a retrospective, longitudinal cohort study in the setting of a Middle-European secondary/tertiary referral center (project name: SolutionX). After informed and written consent, consecutive patients are recruited by a single investigator (M.S.), and all RA patients recruited between September 27, 2017 and April 29, 2019, were included. A positive vote was obtained from the ethical committee of the Medical University of Innsbruck (September 15, 2017, AN 2017-0041 317/4.18). Less than 1% of the patients denied recruitment or could not be recruited because of a psychiatric disease.

The chart review was performed following the STROBE recommendations for cohort studies. Data were selected from the physician's reports stored in the hospital information system (Cerner), if follow-up data were available. The Disease-Activity-Score-28 (DAS28) was calculated using the Clinical Disease Activity Index (CDAI), together with the erythrocyte sedimentation rate (ESR). Medication was classified according to the Anatomical Therapeutical Chemical (ATC) Classification System of the World Health Organization Collaborating Center for Drug Statistics Methodology (WHOCC). Medication was sorted as prescribed daily, weekly, monthly, every few months, and on demand. The absolute number of drugs with two or more effective ingredients was counted once, and then listed in the different ATC classifications of their active agents. In case there was no information about the frequency of intake, the frequency given in the recommendation was used. Local treatments, Chinese and other herbs, homeopathy, and micronutrients were not considered. Obesity was defined using the body mass index (BMI) >30 kg/m^2^, and anemia was defined as hemoglobin (Hb) of <12 g/l in female and <13 g/l in male patients.

After pseudonymization, data were analyzed using the SPSS program (Version 26, October 2019, IBM). Descriptive statistics included means and standard deviations as well as frequencies of different characteristics. Box plots were used to visualize comparisons between groups. The Shapiro–Wilk-test was used to test for normal distributions to decide for further test choices. To compare groups, the Wilcoxon test was used for not normal distributed dependent variables and the Mann–Whitney-*U*-test for independent not normal distributed variables to detect significant increases or decreases in the number of medications between first and last visits for different ATC-Groups and men and women, as well as estimate the significance in differences of gender. For the estimation of possible correlations between the number of medications and factors such as age, disease duration, and -activity, comorbidities and RF, the Pearson correlation coefficient (PCC), and for comparisons between different age groups, the Kruskal–Wallis-Test was used, with additional *post hoc* analysis using Dunn–Bonferroni-tests to exclude possible confounders during the test.

Assessments of the risk of bias to the manuscript were low for patients' selection (>98% of consecutive patients were included), there was no relevant performance or detection bias (with retrospective design). There may be an attrition bias (as patients may have switched to another rheumatologist outside the hospital—with affected the study only if it was the last visit, the manuscript, however, only described the visits in this hospital).

Anonymized data are available by the authors on request.

## Results

### Polypharmacy in Rheumatoid Arthritis Literature

A total of nine studies are included into this review (Figure 1, Table 1). Overall, polypharmacy is common and considerable in RA-patients (6). Polypharmacy has been associated with age, female gender, multimorbidity, disease activity, disease duration, and functional impairment, resulting in a higher risk of hospital admissions (20, 23).

In 24.446 RA-patients from these studies, treatment included an average of 5.3 medications (Table 2). Some of these studies showed that 45.1% of RA-patients have additional comorbidities, and 61.6% of all RA-patients are affected with polypharmacy (Table 2). Polypharmacy was significantly associated with comorbidities and the use of corticosteroids, MTX and bDMARDs (22). Additional aspects were that polypharmacy had a negative impact on health-related quality of life (19), is common also in hospitalized RA-patients (16) with specific RA-medications increasing between 1978 and 1995 (17). RA-patients older and younger than 65 years are treated differently (18), and polypharmacy is associated with drug-related problems [Odds Ratio = 2.96 (1.48–5.91); *p* = 0.003] (21). Besides, a non-compliance rate of 7.6% (drugs being “not taken or administered at all”) is reported with a correlation between non-compliance and polypharmacy (*p* = 0.027) (21).

**Table 2:** Polypharmacy, total number of medications, and frequency of prescribed RA-related medications (DMARDs, glucocorticoids and NSAIDs) in the literature.

**Number of patients [n]**	**Polypharmacy [%]**	**Number of medications**	**DMARDs [% of patients]**	**csDMARD [%]**	**bDMARD [%]**	**Glucocorticoids [%]**	**NSAIDs [%]**	**References**
348	69.5 % (>3)	5.4 (2.4 for RA)	86.8	MTX: 56.3 %	N/A	31.3	19.5	(6)
54	44.4 %	N/A	N/A	N/A	N/A	N/A	N/A	(19)
1,101	N/A	5.2	79	45 % Mono 26 % Double 8 % Triple	22	16	N/A	(20)
200	64.5 %	5.5	94.5	59 % Mono 26.5% Double 7 % Triple MTX: 67 %	1	50	28.5	(21)
792	67.9 % (>5)	5.5 (2.8 for RA)	N/A	90.9 % MTX: 68 %	35.7	47	9.1	(22)
22,005	N/A	5 for others	N/A	N/A	N/A	38	N/A	(23)
			82.5 ± 5.4	84.8 ± 6.1 %	25.2 ± 10.2	37.3 ± 5.1	14.7 ± 7.3	

*N/A, not assessed. Last row mean ± SD, standard deviation, weighted for number of patients*.

### Comorbidities and Side-Effects of RA as Cause of Polypharmacy in the Literature

A mean of 2.8 comorbidities and side effects of RA is reported in 2 studies, and considered responsible for polypharmacy and drug-to-drug interactions (20, 22). More specifically, increasing numbers of comorbidities have been related to the number of drugs (20), with a significant correlation with a standardized regression weight of 0.54 in another study (6). Others describe a correlation between the number of medications and the Rheumatic Disease Comorbidity Index (RDCI), one of various implemented indexes to account comorbid illnesses (23). These authors discuss a possible effect of the severity of comorbidities, as severe diseases may need more medications.

### Adverse Events and Polypharmacy in the Literature

Reporting of adverse events widely varies between the studies. Adverse reactions have been described in 38.8 % of RA-patients, most of them associated with DMARDs, even associated with polypharmacy (21). Another study reported a “non-linear association” between the number of medications and acute hospitalisations, especially for those patients taking more than 10 drugs (20). 44.5% of these hospitalisations happened due to a possible severe adverse event (SAE), and a RA-specific drug was involved in 51.9% of these patients. An increased rate of SAEs was reported together with an increased number of medications, with infection as the most common SAE. The authors' calculation provides an Hazard ratio (HR) of 1.13 as additional risk for SAEs per drug (23).

### Polypharmacy in Rheumatoid Arthritis Cohort

For this Middle-European longitudinal observational study, datasets from the first and the last visit at the rheumatological outpatient clinic (with 6.78 ± 5.42 years apart) are available for 175 RA-patients. Depending on the definition used for polypharmacy (*n* ≥4, ≥5, or ≥6 daily, regular medications), 26.3, 21.1, 14.9% of patients are affected by polypharmacy at the first visit and 45.1, 33.7, and 28.0% of the patients at the last visit, respectively. These percentages for polypharmacy increase from the first to last visit independently from the definition applied. Accordingly, the average number of medications increases from 3.5 ± 2.9 medications at the first to 6.6 ± 3.5 medications at the last visit.

At first visit, women took more drugs than men (3.8 ± 2.9 vs. 2.9 ± 2.8 medications, respectively; *p* = 0.022, using the Wilcoxon test). At last visit, the number of medications increased both for women (to 6.8 ± 15.2; *p* = 0.001, Mann–Whitney-*U*-Test) and men (to 6.2 ± 12.3; *p* = 0.001, Mann–Whitney-*U*-Test), but no longer differed significantly between women and men.

**Figure 2:**
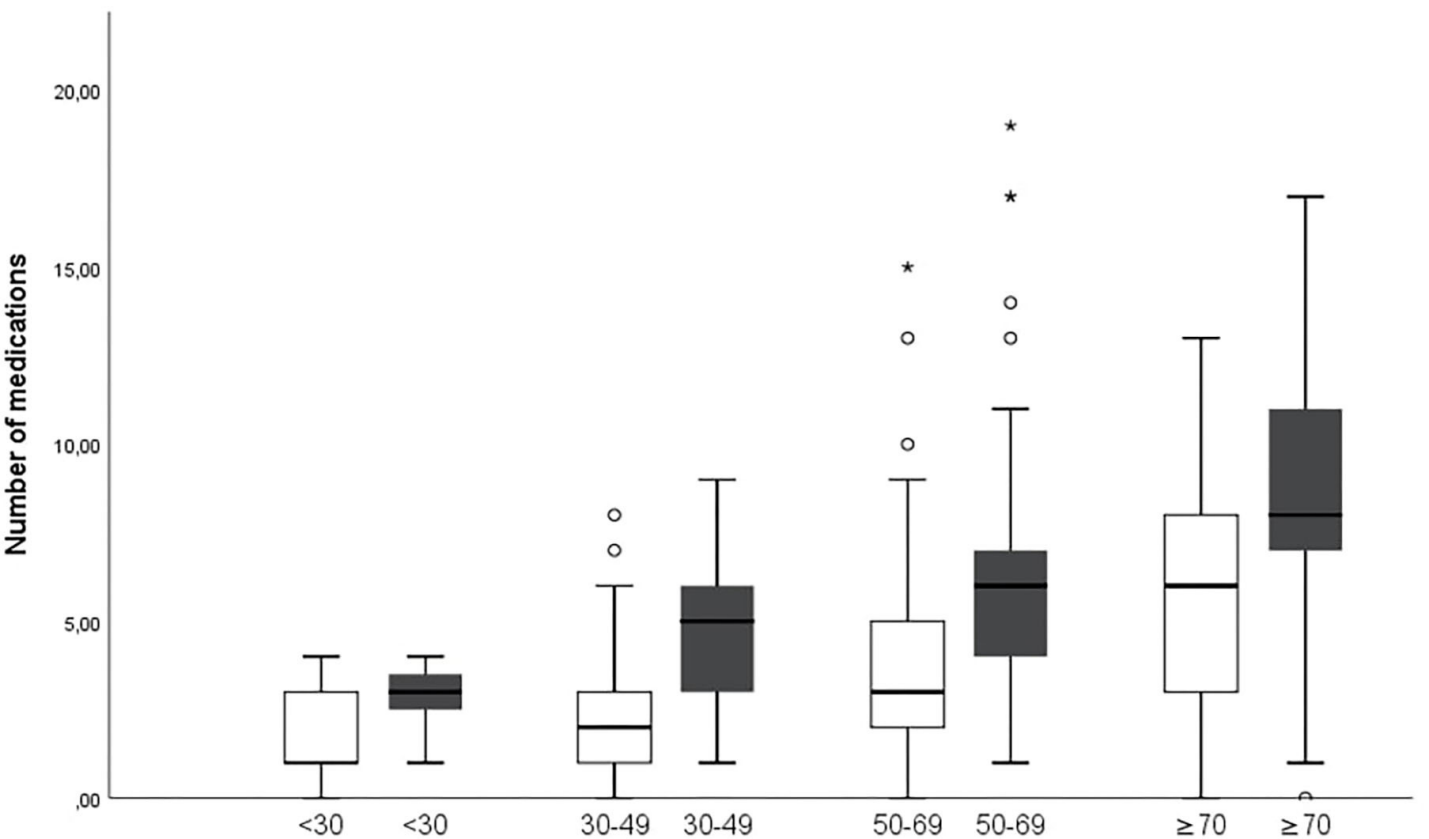
Numbers of medications increase in age groups over 30 years between first (white) and last (gray) visit (*p* = 0.001 for age groups 30–49 and 50–69 years and *p* = 0.014 for age group ≥70 years. *Describes major statistical outliers outside of three times the interquartile range. The symbol is used by SPSS as outlined in the methods section.

The number of drugs increases from first to last visit in all age groups over 30 years (Figure 2). Also, the number of medications correlates with age both at the first visit and the last visit [Pearson Correlation Coefficient (PCC) = 0.457 and 0.460, respectively; *p* = 0.001]. The correlation holds true when male and female gender are analyzed separately, the number of medications correlated with age both in female patients at the first and last visit (PCC = 0.471 and 0.467, respectively; *p* = 0.001), and in male patients (PCC = 0.467 and 0.450, respectively; *p* = 0.002).

### Analysis of ATC-Coded Medication Related to Comorbidities in Rheumatoid Arthritis Cohort

Sorted by the group of ATC codes, medications of 14 ATC-groups and specific classes were taken more frequently at last visit (Table 3). Not all of them were directly related to RA. RA-specific medications include immunosuppressants of the ATC-group L04, hydroxychloroquine, sulfasalazine and glucocorticoids. The number of the RA-specific medications increased from 0.7 (19.7% of all 3.5 medications) at the first visit to 2.4 (36.1% of all 6.6 medications) at the last visit. Analgesics add another 1.0 medications (27.4% of all medications) at the first visit and 0.8 (12.4% of all medications) at the last visit.

**Table 3:** Patient's demographics, disease characteristics and medications at first and last visit (including on request medication, descriptive statistics include means, and standard deviations; percentages in parentheses calculated from total number).

	**First visit**	**Last visit**	***p*-values**
Female gender [%]	73.1	73.1	n.s.
Age [years]	54.5 ± 14.9	61.5 ± 14.4	0.001^**^
Current Smoker [%]	19.4	18.3	n.s.
-Ex-smoker [%]	9.1	14.3	n.s.
Alcohol, occasionally [%]	33.1	45.7	n.s.
Disease duration [months]	32.9 ± 76.3	114.6 ± 101.9	0.001^**^
CDAI	12.7 ± 9.8	5.6 ± 6.9	0.001^**^
DAS28	3.5 ± 1.3	2.7 ± 4.8	0.001^**^
Rheumatoid factor [U/L]	146.2 ± 361.8	77.8 ± 159.7	n.s.
No. of medications	3.5 ± 2.9	6.6 ± 3.5	0.001^**^

** *Highly significant with p < 0.01*.

At the initial visit, 83.4% of all patients showed any comorbidity in addition to RA (Table 4). This percentage further increased during the following 7 years to 96% of all patients having at least one comorbidity or side-effect of RA. The number of all different types of comorbidities increased, except anemia was reduced from 8.0 to 4.6% during follow-up.

**Table 4:** Number of comorbidities at the first visit and months later, assorted to the ATC-group of medication that they are requiring.

**Comorbidity**	**ATC-Code**	**First visit %**	**Last visit**	***p*-value**
Anemia	B03	8.0	4.6	n.s.
Cardiovascular morbidities	C	28.0	47.4	0.001^**^
Diabetes mellitus	A10	6.9	10.9	0.035^*^
Lung diseases	R	2.9	4.6	n.s.
Tendon rupture	M01/M02	2.3	10.9	0.001^**^
Osteopenia/osteoporosis	M05	13.7	41.1	0.001^**^
Osteoarthritis	M01/02	64.0	90.3	0.001^**^
Eye involvement	S01	5.1	12.6	0.002^**^
Thyroid disease	H03	9.1	12.6	n.s.
Muscular disbalances/back pain	M	18.1	33.0	0.001^**^
Neoplasms	L01/L02	9.7	18.3	0.001^**^

*Anemia is defined as Hb<12 for female and Hb<13 for males*.

* *significant with p < 0.05*,

** *highly significant p < 0.01*.

At the first visit, 32% of the patients had a single comorbidity, exceptions occurred with one exemplary patient who presented with 7 different comorbidities beside RA at first visit. Only 6.78 years later, most of the patients (25.1 %) had 4 comorbidities.

Comorbidities and RA side-effects are listed in Table 5 according to the ATC-list. Therefore, osteoarthritis is listed despite being a possible complication of RA after long-standing disease. Other frequent comorbidities involve the cardiovascular system. Accordingly, medications of the ATC-group for cardiovascular medication, as well as antithrombotic medications increased. For example, the number of patients suffering from arterial hypertension increased from 18.3 to 34.9% during follow-up. Also, the number of patients with muscular dysbalances more than doubled from 15.4% at the first to 28% at the last visit. Most comorbidities and RA-related side-effects increased. Using an increasing number of antianaemic agents (ATC-code B03, as numbered in Table 5), only anemia was less frequent at the last compared to the first visit (with 8.0 vs. 4.6%, respectively, Table 4).

**Table 5:** Percentages of ATC-coded medication groups at first and last visit (in alphabetical order).

**ATC code**	**ATC group**	**% first visit**	**% last visit**	**p-value**
A	Alimentary and metabolism	54.3	79.4	0.001^**^
A11/A12	Vitamins and trace elements	28.6	62.3	0.001^**^
B1	Antithrombotic	13.7	25.1	0.001^**^
B03	Antianaemic	14.3	62.9	0.001^**^
C	Cardiovascular	28.6	44.6	0.001^**^
G	Genito-urinary system	1.7	6.3	0.011^**^
H02AB	Glucocorticoids	33.1	4	n.s.
H03	Thyroid gland	20.0	26.3	0.005^**^
J	Antibiotics	1.7	1.7	n.s.
L04	Immunosuppressants	22.9	79.4	0.001^**^
M	musculoskeletal	60.0	64	n.s.
-M01	NSAIDs (regular and on request)	54.9	49.1	n.s.
	NSAIDs (regular intake only)	37.1	12.6	0.001^**^
-M03	Muscle relaxants	0.6	0	n.s.
-M04	Gout	1.7	6.3	0.011^*^
-M05B	Bone diseases	9.1	18.3	0.002^**^
N	Nervous system	20.0	27.4	0.042^*^
N02	Analgesics	9.1	14.9	n.s.
P01	Antimalaria	4	13.1	0.001^**^
R	Respiratory	1.1	5.7	0.011^*^
V	Various	1.1	6.9	0.004^**^

*n.s., not significant*;

* *significant p < 0.05*;

** *highly significant p < 0.01*.

**Figure 3:**
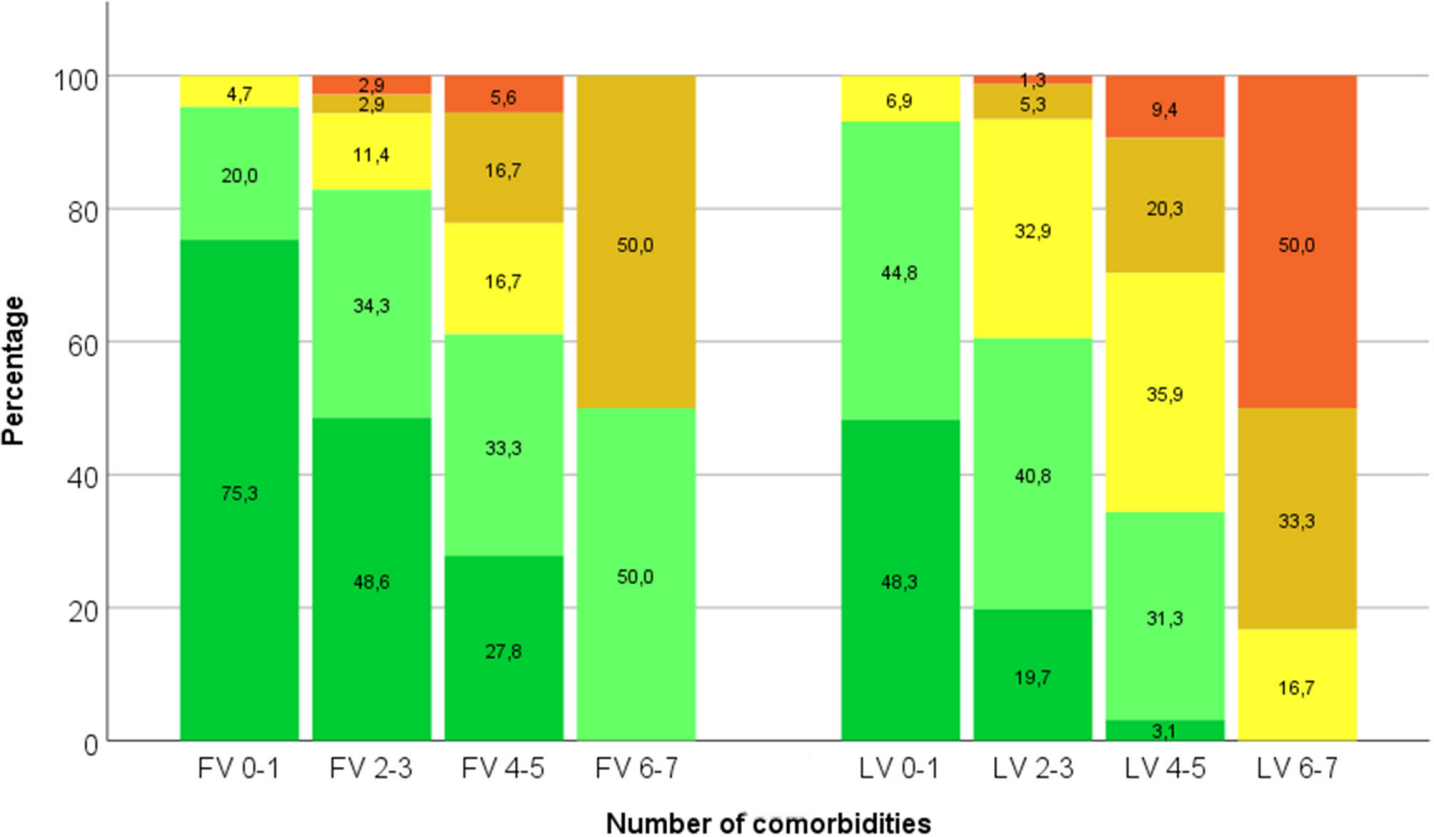
Percentages of patients with increasing number of medications (grouped into ≤3—dark green–, 4–6—light green–, 7–9—yellow–, 10–12—amber–, and >12—red–) depend on number of comorbidities (0–1, 2–3, 4–5, and 6–7) both at first visit (FV) and last visit (LV).

A linear correlation exists between the number of medications and the number of comorbidities and side-effects at the first visit (PCC = 0.458; *p* < 0.001; Figure 3). At the last visit, this linear correlation is even more prominent with a relevant PCC of 0.599 (*p* < 0.001).

### Other Potentially Underlying Factors of Polypharmacy in Rheumatoid Arthritis Cohort

Further analysis was performed for a possible correlation between the number of medications with disease activity, frequency of rheumatologic visits, rheumatoid factor and disease duration. These parameters did not correlate with polypharmacy (data not shown).

## Discussion

This observational study shows polypharmacy (defined as ≥5 medications) in 33.7% of RA-patients, especially in female patients older than 50 years. This number is low in comparison with current literature, reporting polypharmacy in as many as 44.4 to 67.9% of all RA-patients (19, 21, 22). The reason for this difference may be differences of the insurance systems, or the fact that definitions used for polypharmacy are still not consistent (2, 6). In the systematic literature review, the definition of more than 5 drugs was applied in one trial (22), the definitions of more than 3 drugs in another one (6), while all other studies did not provide a specific definition used. In order to make observations and documentations more comparable throughout the literature, a consensus will be helpful for the future. Nevertheless, the average patient had more medications in this cohort compared to the literature (6.6 vs. 5.3, respectively). This indicates different subgroups of patients with more comorbidities and up to 19 medications.

The approach of this study to use ATC-codes for analyses of medication groups is new and has not been applied for the approach to polypharmacy in RA so far. Indeed, ATC-coding does not only allow the comparison between disease-specific treatment and treatment of comorbidities, but also comparison of polypharmaceutical aspects during follow-up. Using the ATC-codes may reduce a possible reporting bias. This can be an advantage for future studies, as new medications like tsDMARDs and bDMARDs can be attributed to existing ATC-codes for comparisons independent from the specific drugs used. Whereas, some newer tsDMARDs and bDMARDs are used on a daily basis, others are applied only every other week or month. 3.4% of RA-patients had more than 4 medications on a weekly basis, although a majority of patients consider “forgetfulness” as an explanation for non-compliance in weekly dosing (24).

All ATC-assorted groups of medications were prescribed more frequently at the last visit compared to the first visit. However, taking a closer look on the different subgroups, major increases were observed for immunosuppressants (L04), treatments for osteopenia/osteoporosis (M05B), and cardiovascular treatment (C). Only the number of analgesics like NSAIDs (M01) was reduced, and more than two thirds of patients with a prescribed NSAID at the first visit did not need further regular prescriptions. These observations went along with the increasing number of medications (from 3.5 to 6.6 over 6.8 years, with 0.9 newly prescribed medications per year of observation). With about 5.3 prescribed medications per patient, the mean number of medications in the few analyzed studies of the SLR was still only slightly lower than at the last visit in our cohort. Only the use of NSAIDs was reduced, in accordance with the intensified treatment for RA. Whereas, at the first visit women took more medications than men, both women and men had more medications at the last visit. Also in the SLR, polypharmacy is reported to be associated with age, whereas results are inconclusive for the gender association (6, 20, 23).

The increasing number of medications strongly correlates with the number of comorbidities (Figure 3). This observation is expected but becomes relevant at the last visit with 95% of the RA-patients suffering from one or more diseases other than RA. Almost every RA-patient needs co-medication together with the RA-treatment, thus easily facing the problem of polypharmacy. As at the same time polypharmacy is suspected as a risk factor for mortality and morbidity, polypharmacy is therefore recommended to be avoided especially in the elderly (1). In this Middle-European cohort, RA-patients over 70 years of age had 8.6 medications if they had already fulfilled the criteria of polypharmacy with 5.8 medications at the first visit. Indeed, the number of medications correlated with age groups. These findings correspond to the literature in general but could be specifically confirmed for RA-patients in this cohort now. Taken together, comorbidities requiring additional medication can be related to the disease course itself or to RA-treatment (for example arterial hypertension after NSAIDs). Comorbidities, however, should not lead to hesitance of prescribing DMARDs to achieve the treat-to-target goals. Certainly not age itself but the comorbidities make a difference between the vital and the frail patient, as mentioned by many rheumatologists (25). Furthermore, comorbidities are one of the most frequent reasons, that cause a difficult-to-treat RA, alongside with extra-articular manifestations (26). In this Middle-European cohort the number of prescribed medications did not correlate with disease activity and disease duration, which had been proposed earlier in other studies (6, 20).

The question, how much the intake of multiple medications with possible interactions harms the patient more than provides a benefit, and reduces the patients' compliance, is still unanswered. Bechman et al. (23) addressed two aspects of polypharmacy in his paper. The aspect of serious adverse events (SAE) could not be assessed in the Innsbruck cohort, as SAEs did not occur within the observational period. A certain bias because of admission to different local hospitals cannot be excluded. Occurrence of SAEs is certainly another important aspect of polypharmacy. The EULAR good response rate was observed to be lower with polypharmacy in his work. In the Innsbruck cohort, disease activity scores and polypharmacy showed no correlation and further detailed study would be needed to assess individual improvements compared to number of medications prescribed.

The most important limitation of this study is its retrospective design with incomplete datasets (resulting e.g., in heterogeneity of times from first to last visit) and the relatively small sample size. Also, more detailed information on the type of increase in number of medications (early or late, linear or logarithmic) could not be answered in this retrospective study. In the literature review, one study included only 50% of the 295 patients with RA (18), and not all studies provide exact numbers of patients with comorbidities. Second, there is no consistent definition which comorbidities to be assessed in clinical studies with RA. In this Middle-European study osteoarthritis is considered as comorbidity, although osteoarthritis could also be secondary to RA. Also, anemia can be related to RA itself, to its medication or occur independent from both (but still is summarized as ATC-code B03). Age and the number of comorbidities certainly are confounders for this study. With this approach, it cannot be excluded, that the number of medications does not only correlate with the number of comorbidities, but purely depends on the increased age. Furthermore, the female gender could be a confounder because of similar age changes. Concerning the SLR, the exclusion of Embase and a biased rob of the studies have to be considered as additional limitations.

Improved studies on polypharmacy have to rely on detailed data sets, including all parameters possible relevant for assessment of risk factors and consequences of polypharmacy. Disease-specific treatment may prevent additional prescription of analgesics, thus avoiding unnecessary polypharmacy and further supporting the strategy of T2T (27). In RA-patients, knowledge about the underlying diagnosis, risk of disease complications and comorbidities will be critical to improve patients' adherence, especially in aged women with comorbidities. If the patient needs more pain medication than expected, diagnosis and treatment should be reconsidered. Drug indications, contraindications, and doses have to be re-evaluated on a regular basis, to assure an optimal pharmacological treatment without unnecessary polypharmacy.

## Conclusion

In rheumatoid arthritis, polypharmacy affects more than a third of RA-patients and increases with age and number of comorbidities. Only a few RA-studies focus on polypharmacy in the literature so far. In future studies the definitions of polypharmacy should be reported, and a consensus be reached on the most relevant definition of polypharmacy.

## Data Availability Statement

The raw data supporting the conclusions of this article will be made available by the authors, without undue reservation.

## Ethics Statement

The studies involving human participants were reviewed and approved by Ethics committee of the Medical University Innsbruck, AN 2017-0041 370/4.18. The patients/participants provided their written informed consent to participate in this study.

## Author Contributions

JD and RM: data collection. JD and SM: statistical analysis. JD and MS: concept and design of the study, drafting the manuscript. All authors: final corrections and approval.

## Conflict of Interest

The authors declare that the research was conducted in the absence of any commercial or financial relationships that could be construed as a potential conflict of interest.

## References

[1] Hajjar ER Cafiero AC Hanlon JT. Polypharmacy in elderly patients. Am J Geriatr Pharmacother. (2007) 5:345–51. 10.1016/j.amjopharm.2007.12.002 18179993

[2] Masnoon N Shakib S Kalisch-Ellett L Caughey GE. What is polypharmacy? A systematic review of definitions. BMC Geriatr. (2017) 17:230. 10.1186/s12877-017-0621-2 29017448 PMC5635569

[3] Massey EB Simpson TW Ariail JC Simpson KN Bushardt RL. Polypharmacy: misleading, but manageable. Clin Interv Aging. (2008) 3:383–9. 10.2147/CIA.S2468 18686760 PMC2546482

[4] Pflugbeil S Böckl K Pongratz R Leitner M Graninger W Ortner A. Drug interactions in the treatment of rheumatoid arthritis and psoriatic arthritis. Rheumatol Int. (2020) 40:511–21. 10.1007/s00296-020-04526-3 32052146

[5] Fialová D Topinková E Gambassi G Finne-Soveri H Jónsson PV Carpenter I. Potentially inappropriate medication use among elderly home care patients in Europe. JAMA. (2005) 293:1348–58. 10.1001/jama.293.11.1348 15769968

[6] Treharne GJ Douglas KM Iwaszko J Panoulas VF Hale ED Mitton DL. Polypharmacy among people with rheumatoid arthritis: the role of age, disease duration and comorbidity. Musculoskeletal Care. (2007) 5:175–90. 10.1002/msc.112 17623274

[7] Kronzer VL Crowson CS Sparks JA Myasoedova E Davis JM. Comorbidities as risk factors for rheumatoid arthritis and their accrual after diagnosis. Mayo Clin Proc. (2019) 94:2488–98. 10.1016/j.mayocp.2019.08.010 31759675 PMC6907158

[8] Myasoedova E Davis J Matteson EL Crowson CS. Is the epidemiology of rheumatoid arthritis changing? Results from a population-based incidence study, 1985-2014. Ann Rheum Dis. (2020) 79:440–4. 10.1136/annrheumdis-2019-216694 32066556 PMC7085464

[9] Wolfe F Mitchell DM Sibley JT Fries JF Bloch DA Williams CA. The mortality of rheumatoid arthritis. Arthritis Rheum. (1994) 37:481–94. 10.1002/art.1780370408 8147925

[10] Gwinnutt JM Symmons DPM MacGregor AJ Chipping JR Marshall T Lunt M. Have the 10-year outcomes of patients with early inflammatory arthritis improved in the new millennium compared with the decade before? Results from the norfolk arthritis register. Ann Rheum Dis. (2018) 77:848–54. 10.1136/annrheumdis-2017-212426 29475855 PMC5965352

[11] Mitchell DM Spitz PW Young DY Bloch DA McShane DJ Fries JF. Survival, prognosis, and causes of death in rheumatoid arthritis. Arthritis Rheum. (1986) 29:706–14. 10.1002/art.1780290602 3718563

[12] Radner H Smolen JS Aletaha D. Impact of comorbidity on physical function in patients with rheumatoid arthritis. Ann Rheum Dis. (2010) 69:536–41. 10.1136/ard.2009.118430 19825848

[13] Michaud K Wolfe F. Comorbidities in rheumatoid arthritis. Best Pract Res Clin Rheumatol. (2007) 21:885–906. 10.1016/j.berh.2007.06.002 17870034

[14] Zelko E KlemencKetis Z TusekBunc K. Medication adherence in elderly with polypharmacy living at home: a systematic review of existing studies. Mater SocioMed. (2016) 28:129–32. 10.5455/msm.2016.28.129-132 27147920 PMC4851507

[15] Fincke BG Miller DR Spiro A III. The interaction of patient perception of overmedication with drug compliance and side effects. J Gen Intern Med. (1998) 13:182–5. 10.1046/j.1525-1497.1998.00053.x 9541375 PMC1496921

[16] Friesen WT Hekster YA van de Putte LB Gribnau FW. Cross-sectional study of rheumatoid arthritis treatment in a university hospital. Ann Rheum Dis. (1985) 44:372–8. 10.1136/ard.44.6.372 3874606 PMC1001655

[17] Bergström U Book C Lindroth Y Marsal L Saxne T Jacobsson L. Lower disease activity and disability in Swedish patients with rheumatoid arthritis in 1995 compared with 1978. Scand J Rheumatol. (1999) 28:160–5. 10.1080/03009749950154239 10380838

[18] Juby A Davis P. An evaluation of the impact of seniors on a rheumatology referral clinic: demographics and pharmacotherapy. Clin Rheumatol. (2011) 30:1507–9. 10.1007/s10067-011-1845-8 21935585

[19] González-Gamboa LM Barocio-Ramírez AK Rocha-Muñoz AD de Santos-Ávila F Meda-Lara RM González-López L. Disease activity score on 28 joints and polypharmacy are independent predictors for health-related quality of life evaluated by INCAVISA in patients with rheumatoid arthritis. J Clin Rheumatol. (2016) 22:399–404. 10.1097/RHU.0000000000000463 27870761

[20] Filkova M Carvalho J Norton S Scott D Mant T Molokhia M. Polypharmacy and unplanned hospitalizations in patients with rheumatoid arthritis. J Rheumatol. (2017) 44:1786–93. 10.3899/jrheum.160818 28966210

[21] Ma SN Huri HZ Yahya F. Drug-related problems in patients with rheumatoid arthritis. Ther Clin Risk Manag. (2019) 15:505–24. 10.2147/TCRM.S194921 30962689 PMC6432894

[22] Gomides APM Albuquerque CP Santos ABV Amorim RBC Bértolo MB Júnior PL. High levels of polypharmacy in rheumatoid arthritis-A challenge not covered by current management recommendations: data from a large real-life study. J Pharm Pract. (2019). 10.1177/0897190019869158. [Epub ahead of print]. 31451091

[23] Bechman K Clarke BD Rutherford AI Yates M Nikiphorou E Molokhia M. Polypharmacy is associated with treatment response and serious adverse events: results from the british society for rheumatology biologics register for rheumatoid arthritis. Rheumatology. (2019) 58:1767–76. 10.1093/rheumatology/kez037 30982886

[24] Azevedo R Bernardes M Fonseca J Lima A. Smartphone application for rheumatoid arthritis self-management: cross-sectional study revealed the usefulness, willingness to use and patients' needs. Rheumatol Int. (2015) 35:1675–85. 10.1007/s00296-015-3270-9 25903352

[25] Nawrot J Boonen A Peeters R Starmans M Van Onna M. Rheumatologists' views and experiences in managing rheumatoid arthritis in elderly patients: a qualitative study. J Rheumatol. (2018) 45:590–4. 10.3899/jrheum.170773 29449497

[26] Roodenrijs NMT De Hair MJH Van Der Goes MC Jacobs JWG Welsing PMJ Van Der Heijde D. Characteristics of difficult-to-treat rheumatoid arthritis: results of an international survey. Ann Rheum Dis. (2018) 77:1705–9. 10.1136/annrheumdis-2018-213687 30194273

[27] Solomon DH Bitton A Katz JN Radner H Brown EM Fraenkel L. Review: treat to target in rheumatoid arthritis: fact, fiction, or hypothesis? Arthritis Rheumatol. (2014) 66:775–82. 10.1002/art.38323 24757129 PMC4012860

